# The spino-bulbar-cerebellar pathway: organization and neurochemical properties of spinal cells that project to the lateral reticular nucleus in the rat

**DOI:** 10.3389/fnana.2015.00001

**Published:** 2015-01-22

**Authors:** Zilli Huma, David J. Maxwell

**Affiliations:** Spinal Cord Group, Institute of Neuroscience and Psychology, College of Medicine, Veterinary Medicine and Life Sciences, University of GlasgowGlasgow, UK

**Keywords:** spinal cord, motor control, cerebellum, neurotransmitters, neuroanatomy

## Abstract

In addition to classical spinocerebellar pathways, the cerebellum receives information from the spinal cord indirectly via spino-bulbar-cerebellar systems. One of the structures in this pathway is the lateral reticular nucleus (LRt). We performed series of experiments to investigate the organization and neurotransmitter content of spinoreticular tract (SRT) neurons in the lumbar spinal cord that project to the LRt. Three rats received injections of the b subunit of Cholera toxin (CTb) or Fluorogold (FG) within the left and right LRt. The majority of SRT cells (56–61%) were found within the contralateral medial intermediate gray matter where small numbers (7–10%) of double-labeled cells were also present on both sides of the cord. Six rats received unilateral spinal injections of CTb to label spinal projections to the LRt. Injections of FG were made also into the anterior lobe of the cerebellum to label LRt pre-cerebellar neurons. Terminals were found mainly ipsilateral to spinal injection sites within the central and ventrolateral regions of the LRt. Immunocytochemical analysis of SRT terminals revealed that the majority (75%) were contained vesicular glutamate transporter 2 but a minority (20%) contained the vesicular GABA transporter. The inhibitory subpopulation was found to be GABAergic, glycinergic, or contained both transmitters. Inhibitory and excitatory terminals were present within overlapping regions of the nucleus. Most CTb terminals contacting LRt pre-cerebellar neurons were excitatory (80%) whereas a minority were inhibitory and most cells (88%) received contacts from both inhibitory and excitatory terminals. This study shows that SRT axons in the LRt have the capacity to exert direct excitatory and inhibitory actions on LRt pre-cerebellar neurons. Thus spinal cord input has the capacity to facilitate or depress the activity of individual LRt cells which in turn adjust activity in the cerebellum to produce coordinated motor behaviors.

## INTRODUCTION

In addition to the classical direct spinocerebellar pathways, indirect pathways exist that convey information from the spinal cord to the cerebellum (Clendenin et al., 1974a,b; Arshavsky et al., 1984). These pathways consist of SRT neurons that form synaptic connections with pre-cerebellar neurons located in the medullary reticular formation. The principal pre-cerebellar nucleus involved in this spino-bulbar-cerebellar pathway is the LRt which contains neurons that are known to have highly ordered projections to different regions of the cerebellar cortex and deep cerebellar nuclei. (Clendenin et al., 1974c; Künzle, 1975; Matsushita and Ikeda, 1976; Dietrichs and Walberg, 1979; Hrycyshyn et al., 1982; Menétrey et al., 1983; Wu et al., 1999). Lesions of the LRt cause disruption of posture and balance (Corvaja et al., 1977b; Santarcangelo et al., 1981) and the LRt degenerates in spinocerebellar ataxia type 3 (Marcado–Joseph Disease) which is characterized by severe limb and gait ataxia (Rüb et al., 2002) thus confirming its importance in motor integration.

Electrophysiological studies have shown that at least three distinct spinal pathways project to the LRt. These include two pathways that originate from cervical segments, the iFT and the C3–C4 propriospinal system (see Alstermark and Ekerot, 2013). A third pathway, the bVFRT projects from all spinal levels. In addition to these spinal pathways, LRt cells can also be activated from a number of other brain regions including, the trigeminal nerve, the rubrospinal tract, superior colliculus, and motor cortex (see Alstermark and Ekerot, 2013). This pattern of connectivity suggests that the LRt has an important role in coordinating motor activity by integrating information from the spinal cord and various regions of the brain (Corvaja et al., 1977b). Alstermark and Ekerot (2013) have suggested that the cerebellum compares integrated information from the LRt with segregated information conveyed by the classical spinocerebellar pathways in order to adjust errors in motor programs.

In rats and in cats, unilateral injections of retrograde tracer within the LRt label cells at all segmental levels. Most cells are labeled contralateral to LRt injection sites, and in the lumbar cord, are concentrated within laminae VII, VIII, and X (Corvaja et al., 1977a; Hrycyshyn and Flumerfelt, 1981; Menétrey et al., 1983; Shokunbi et al., 1985). Lesion and tracing studies also reveal that spinal neurons form numerous terminations within the LRt (Brodal, 1949; Rajakumar et al., 1992). Degeneration of terminals occurs within the ipsilateral LRt following lesions of the ventrolateral quadrant (Hrycyshyn and Flumerfelt, 1981). These inputs are highly ordered and project to specific regions of the LRt; for example projections from the lumbar cord terminate in the ventral and ventrolateral region of the nucleus whereas those from cervical regions terminate throughout the LRt (Künzle, 1973; Clendenin et al., 1974b; Rajakumar et al., 1992; see also Pivetta et al., 2014). Although there is general agreement about the spinal organization of SRT cells projecting to the LRt, there is contradictory evidence supporting the existence of SRT neurons that project bilaterally to both nuclei. One study reports that there are almost no bilateral projections (Koekkoek and Ruigrok, 1995) whereas another reports that 7% of SRT cells project to both nuclei (Garifoli et al., 2006). An initial aim of the present study therefore was to re-examine the organization of SRT cells in the lumbar spinal cord following bilateral injections of tracer substances.

Electrical stimulation of the lumbar component of the bVFRT not only evokes strong excitatory responses in LRt neurons but also causes profound inhibition (Rosen and Scheid, 1972, 1973a,b; Ekerot, 1990a) and some inhibitory actions are evoked with monosynaptic latencies (Ekerot, 1990b). There is limited evidence for long ascending inhibitory spinal pathways from the spinal cord to the brain but recently the existence of a long inhibitory pathway to the ventromedial medulla was established by Hossaini et al. (2012) in an anatomical study. Pivetta et al. (2014), using viral tracing techniques in genetically modified mice reported that lumbar projections to the LRt are almost exclusively excitatory but noted the existence of sparse inhibitory terminals within the most ventromedial region of the LRt. This finding is somewhat at variance with those reported in the electrophysiological study of Ekerot (1990b) who concluded that excitatory and inhibitory lumbar bVFRT neurones have similar termination areas in the LRt. The principal aim of the present study therefore was to determine if inhibitory and excitatory axon terminals within the LRt arise directly from neurons within the lumbar cord and, if so, to determine if they are segregated anatomically within the LRt. We also wished to determine if inhibitory SRT axons form direct contacts with pre-cerebellar LRt neurons and if excitatory and inhibitory SRT terminations converge on these cells.

## MATERIALS AND METHODS

A series of anatomical tract tracing experiments was undertaken on nine adult male Sprague Dawley rats (250–350 gm Harlan, Bicester, UK). These experiments were conducted according to British Home Office legislation and were approved by the University of Glasgow Ethics Committee for Animal Research. In the first series of experiments bilateral injections were made in the left and right LRt of three animals to label SRT cells in the lumbar spinal cord retrogradely. In the second series, the ventral quadrant of the lumbar cord of six rats was injected with the b subunit of CTb and terminals in the LRt were examined for immunoreactivity for VGLUTs (VGLUT1 and VGLUT2) and the VGAT to identify excitatory and inhibitory terminals, respectively. A final series of experiments was performed where we examined the relationships formed by these axons with pre-cerebellar LRt neurons that project to the anterior lobe of the cerebellum where LRt cells with input from the lumbar component of the SRT are known to form terminations (Alstermark and Ekerot, 2013).

### SURGICAL PROCEDURES

Surgery was performed on animals induced and maintained under general anesthesia with 2–4% Isoflurane in oxygen.

In order to label SRT cells, three adult male Sprague Dawley rats were placed in a strereotaxic frame. Two different retrograde tracers were injected into the left and right LRt. On the left side 200 nl of the b subunit of CTb (Sigma–Aldrich, Co., Poole, UK; 1% in sterile distilled water) was pressure injected at inter-aural co-ordinates -4.8 mm (antero-posterior), +1.8 mm (medio-lateral) and at a dorso-ventral coordinate of -0.4 mm (Paxinos and Watson, 2005). The micropipette was left in place for 5 min to prevent any backflow of the tracer and then emptied by ejecting the remaining CTb on to a cotton bud and cleaned by drawing up sterile distilled water and ejecting it several times. The outer surface of the tip was cleaned with distilled water on a sterile cotton bud. A second burr hole was made on the right side at interaural co-ordinates -4.8 mm (antero-posterior) and -1.8 mm (medio-lateral). The pipette was then filled with 50 nl of 4% Fluorogold (FG) in distilled water (Fluorochrome, LLC, USA) and pressure injected into the right LRt at a dorso-ventral coordinate of -0.4 mm. The pipette was again left in place for 5 min and then removed. The scalp was sutured and animals were allowed to recover.

To label axon terminals projecting to the LRt, six rats were mounted in a spinal frame and the Th13 vertebra was identified by its attachment to the last rib. A small dorsal midline incision extending from Th10 to L3 vertebrae was made. The laminar surface of the second lumbar vertebra was exposed (on the right side of the animal) and a small burr hole was created on the right side adjacent to the midline. A glass micropipette was then inserted to a depth of ∼1.5 mm into the spinal cord at an angle of 15 and 100 nl of CTb was injected by using a Pico–Injector (World Precision Instruments, Sarasota, FL, USA). Following a period of 48 h three of these animals were anesthetized with Isoflurane as described above and placed in a stereotaxic frame. These animals were given stereotaxic injections of FG (50 nl of 4% in sterile distilled water) within the anterior cerebellum via a glass micropipette (inter-aural co-ordinates, dorso–ventral +6 mm, antero–posterior -1.6 mm, and medio-lateral -0.5 mm; Paxinos and Watson, 2005).

All animals were given subcutaneous injections of Rimadyl 0.1 ml/100 gm (Carprofen 50 mg/ml, Pfizer, Dumfries, UK) and 0.15 ml/100 gm Vetergesic (Buprenorphine 0.3 mg/ml, Reckitt Benckiser Healthcare, Dumfries, UK) postoperatively.

### FIXATION

Following a 6 day survival period after the initial surgical procedure, rats were anesthetized with pentobarbitone (1 ml i.p.) and perfused through the left ventricle with mammalian Ringer’s solution followed by one liter of a fixative containing 4% formaldehyde in 0.1 M PB (pH 7.4) at room temperature. Brains and spinal cords were removed and placed in the same fixative overnight except that 30% sucrose was added to the fixative for brains to cryoprotect brain tissue to be sectioned on a freezing microtome.

### INJECTION SITES

To examine LRt injection sites, 100 μm coronal sections of the medulla were sectioned with a freezing microtome (Leica, UK). Alternate sections were reacted for CTb. Sections were incubated in goat anti- CTb for 48 h followed by biotinylated anti-goat IgG for 3 h at room temperature prior to incubation in avidin-HRP for 1 h and hydrogen peroxide plus DAB for a period of approximately 15 min. They were then mounted on gelatin-coated slides, dehydrated, cleared, and a coverslip was applied. Adjacent sections were mounted with anti-fade medium (Vectashield, Vector Laboratories, Peterborough, UK) on glass slides.

Spinal cord segments containing injection sites were sectioned (transverse 50 μm sections) with a Vibratome (Oxford instruments, Technical products international Inc. USA) and reacted to reveal CTb as described above for medullary sections. For animals with FG injections in the cerebellum, the cerebellum was detached from the brainstem, sectioned with a freezing microtome (100 μm coronal sections) and mounted with Vectashield on glass slides.

Injection sites containing CTb were viewed with light microscopy, whilst those containing FG were viewed with epifluorescence. Sections were photographed digitally (AxioVision 4.8 software, Zeiss, Germany) and the location of each injection site was determined from captured digital images with reference to the stereotaxic rat brain atlas of Paxinos and Watson (2005).

### IMMUNOREACTIONS

Immunoreactions were performed on 50 μm coronal Vibratome sections of the spinal cord and medulla according to the details listed in **Table 1**. All sections were treated with an aqueous solution of 50% ethanol for 30 min to enhance antibody penetration. Briefly, sections were incubated in combinations of primary antibodies (see **Table 1**) for 48 h. Sections were then washed several times with 0.1 M PBS that contained 0.3 M NaCl (PBS) before transferring them to combinations of secondary antibodies conjugated to fluorophores for 3 h. After a final wash in PBS, sections were mounted with anti-fade medium, Vectashield (Vector Laboratories, Peterborough, UK) on glass slides.

**Table 1:** Antibodies used in the study.

Experiment	Primary antibody combination	Primary antibody concentration	Supplier	Secondary antibody combination	Secondary antibody concentration	Supplier
1	gt CTb rbt FG	1:5000 1:5000	List (Quadratech), Campbell, CA, USA Chemicon/Millipore, CA, USA	Rh. Red Alexa 488	1:100 1:500	Jackson Immunoresearch, West Grove, PA, USA Molecular probes, Eugene, OR, USA
2A	gt CTb	1:5000	List (Quadratech), Campbell, CA, USA	Rh. Red	1:100	Jackson Immunoresearch, West Grove, PA, USA
	gp VGLUT1&2	1:5000	Chemicon, Harlow, UK	Alexa 647	1:500	Molecular probes, Eugene, OR, USA
	rbt VGAT	1:5000	Synaptic systems, Göttingen, Germany	Alexa 488	1:500	Molecular probes, Eugene, OR, USA
2B	gt CTb	1:5000	List (Quadratech)	Rh. Red	1:100	Jackson Immunoresearch, West Grove, PA, USA
	gp VGLUT1	1:5000	Chemicon, Harlow, UK	Alexa 647	1:500	Molecular probes, Eugene, OR, USA
	rbt VGLUT2	1:5000	Chemicon, Harlow, UK	Alexa 488	1:500	Molecular probes, Eugene, OR, USA
2C	gt CTb	1:5000	List (Quadratech)	Rh. Red	1:100	Jackson Immunoresearch, West Grove, PA, USA
	gp GLYT2	1:10000	Chemicon, Harlow, UK	Alexa 488	1:500	Molecular probes, Eugene, OR, USA
	rbt GAD 65&67	1:1000	Sigma	Alexa 647	1:500	Molecular probes, Eugene, OR, USA
3	gt CTb	1:5000	List (Quadratech)	Rh. Red	1:100	Jackson Immunoresearch, West Grove, PA, USA
	rbt FG	1:5000	Chemicon/Millipore, CA, USA	Alexa 488	1:500	Molecular probes, Eugene, OR, USA
	gpig VGLUT2	1:5000	Chemicon, Harlow, UK	Alexa 647	1:500	Molecular probes, Eugene, OR, USA
	mo VGAT	1:1000	Synaptic systems, Göttingen, Germany	mo Biotin AV-PB	1:500 1:1000	Molecular probes, Eugene, OR, USA

*gt, goat; rbt, rabbit; mo, mouse; gp, guinea pig; FG, Fluorogold; CTb, the b subunit of cholera toxin; VGLUT1, vesicular glutamate transporter 1; VGLUT2, vesicular glutamate transporter 2; VGAT, vesicular GABA transporter; GLYT2, glycine transporter 2; GAD 65+67 = 65 and 67 isoforms of glutamate decarboxylase; Rh. Red, rhodamine red; Mo Biotin AV-PB, mouse biotin, avidin-pacific blue. In Experiment 1A tissue was incubated in a mixture of VGLUT1+2 antibodies.*

### IMAGING AND DATA ANALYSIS

Sections were analyzed with a confocal microscope (LSM 710, Zeiss, Germany).

To examine retrogradely labeled SRT cells, spinal cord sections (three sections from L3, L4, and L5 for each animal) were tile scanned through their full thickness with the confocal microscope at a magnification of x10 (1.4 zoom at 2 μm steps). CTb, FG, and double-labeled cells were plotted and counted by using Neurolucida for Confocal (MBF Bioscience, Colchester, VT, USA) software. The first and last two optical sections were excluded from the analysis to minimize counting errors. The locations of cells were plotted on to outline diagrams. The proportions of cells of each type (ipsilateral, contralateral, and bilateral) were expressed as a percentage which was averaged for the three animals.

To examine terminals in the LRt, tile scans of 5 × 3 image stacks covering the LRt were taken (magnification 40X lens, zoom factor 1.4, each stack consisting of 20 z-steps at 0.5 μm intervals) were made from two sections (ipsilateral LRt) or two sections (contralateral LRt) per animal taken from interaural coordinates a levels -4.92 and -5.40 mm. Single optical sections from image stacks consisting of 20 optical sections were viewed with Neurolucida for Confocal. Initially sections were viewed in the red channel so that only CTb immunoreactivity was visible. Terminals were defined as axonal swellings that ranged between 0.5 and 3 μm in diameter. A grid consisting of 25 μm × 25 μm squares was superimposed upon the individual sections and for each square, the CTb labeled terminal closest to the bottom left corner was marked with a symbol for analysis. This procedure was repeated until the entire LRt had been sampled within each stack. Annotated terminals were subsequently examined in the blue and green channels in order to assess expression of transmitter-related markers. The percentage of terminals expressing each of the markers was then calculated and averaged for the six animals.

Contacts on pre-cerebellar LRt neurons containing FG made by CTb labeled axon terminals were investigated for the presence of VGLUT2 and VGAT immunoreactivity. In total 33 cells were investigated (11 each from animals 1–3). Pre-cerebellar LRt neurons were reconstructed from confocal images with Neurolucida for Confocal and the location of CTb contacts was plotted onto the reconstructions. A contact was defined as a close apposition between a labeled terminal and a cell where no intervening dark pixels were present. The numbers of contacts containing VGLUT2 or VGAT were counted and the contact density of the two types of terminals in contact with cells was expressed as numbers of contacts per 100 μm^2^ units of the neuronal surface area which was calculated using the Neuroexplorer facility of Neurolucida.

Data are expressed as mean ± SD and multiple comparisons were made using ANOVA with *post hoc* Tukey’s correction (*p* < 0.05).

## RESULTS

### RETROGRADELY LABELED SRT CELLS

**FIGURE 1:**
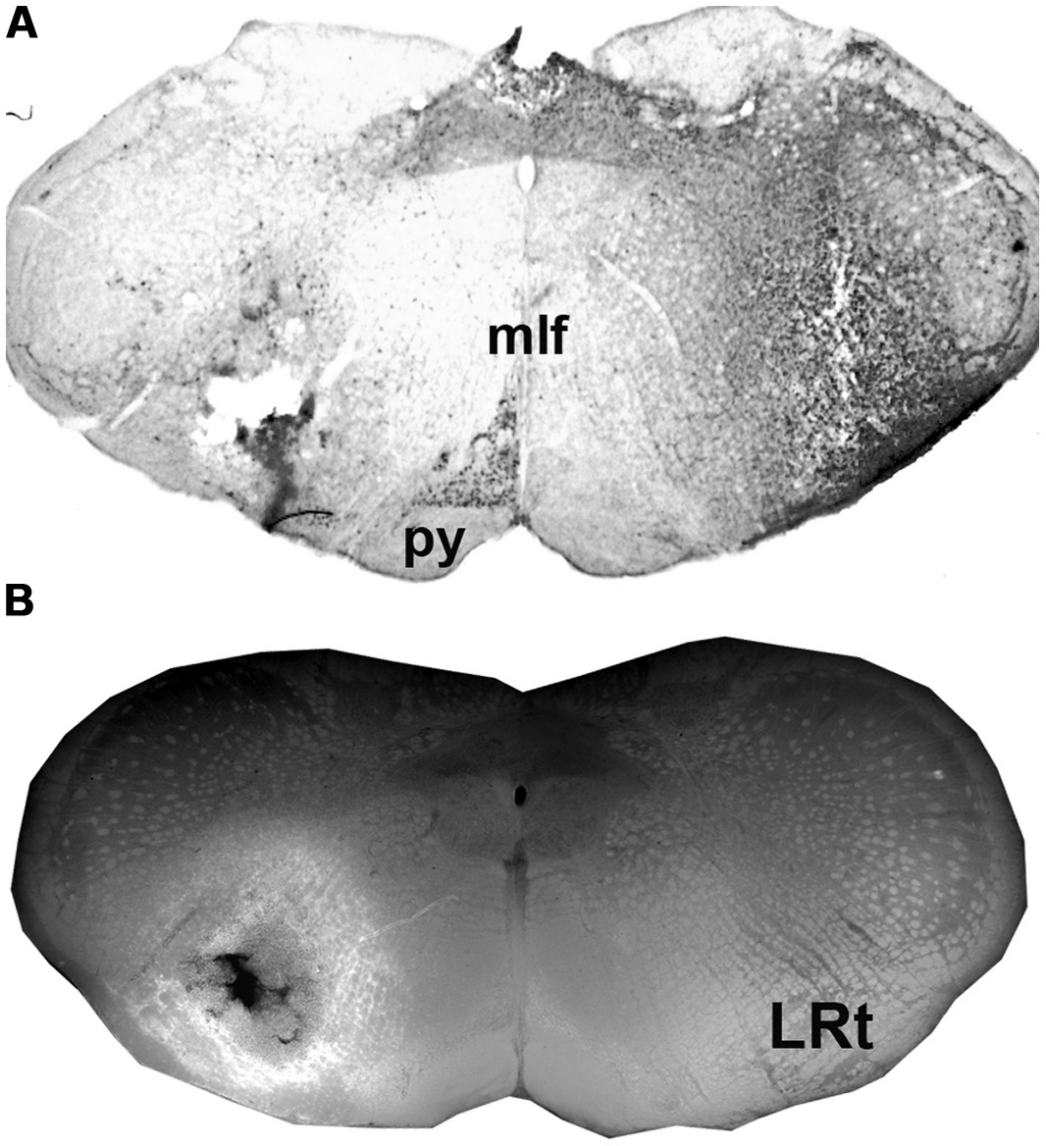
**Injections sites in the medulla.** **(A)** A CTb injection site **(B)** An adjacent section showing a combined dark-field and epifluorescence image showing Fluorogold (FG) within the LRt and surrounding reticular formation. A schematic version of these injections is shown in **Figure 2A**. LRt, lateral reticular nucleus, mlf, medial longitudinal fasciculus, py, pyramid.

**FIGURE 2:**
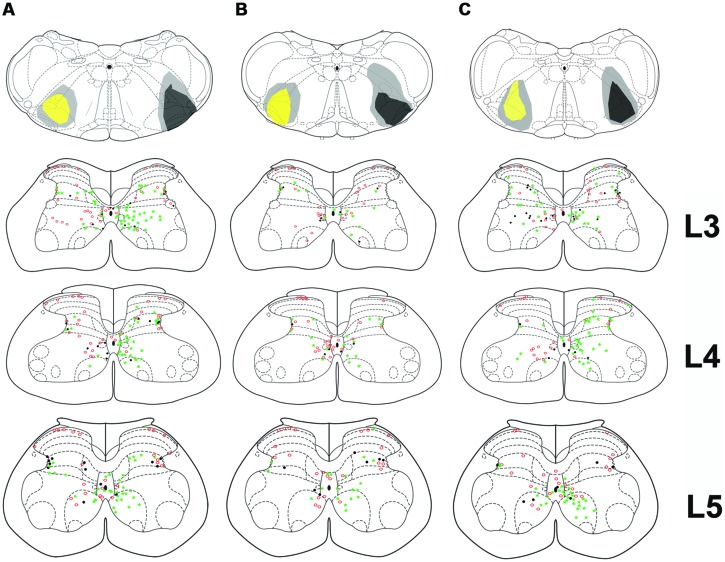
**Bilateral medullary injection sites and distribution of spinoreticular tract (SRT) cells in lumbar segments from 3 animals (A–C).** FG injections are shown as yellow and CTb as black. Diffuse spread of tracer is shown as gray. Distribution of cells for three segments (L3–L5) is shown on schematic diagrams of transverse sections. Each diagram represents a composite analysis of 3 μm × 50 μm sections from the three segments. Green stars, FG labeled cells; Red circles, CTb labeled cells; black dots, cells labeled with both tracers.

**FIGURE 3:**
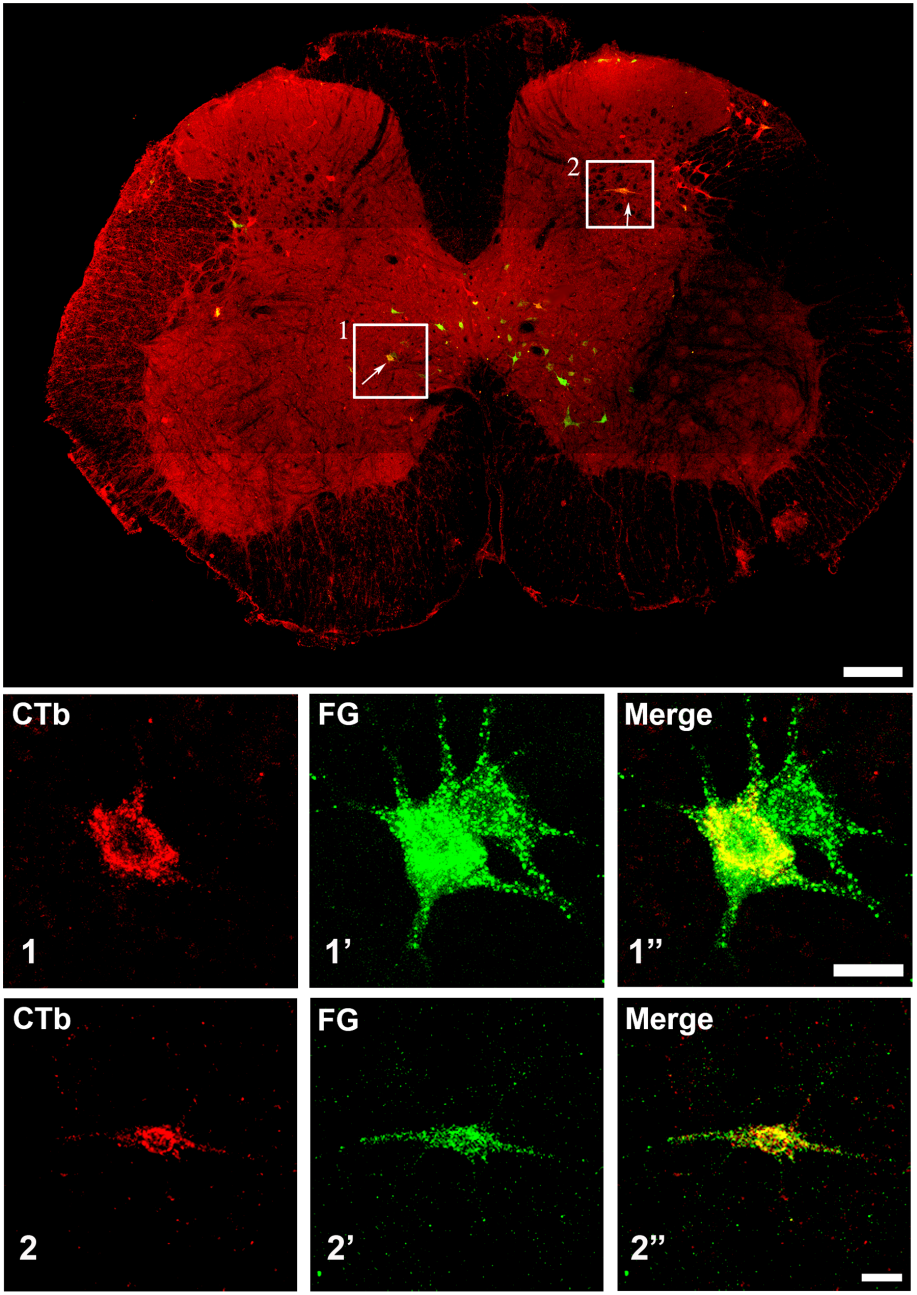
**Confocal microscope images of spinal cells labeled with FG (green) and CTb (red).** The main plate is a tiled image of an entire L3 transverse section. The areas demarcated by boxes 1 and 2 illustrate examples of double labeled cells within laminae VII and V, respectively, and are shown as single optical sections at higher magnification below (1 and 2). Scale bars: Main plate = 200 μm; 1 and 2 = 20 μm.

Injection sites for CTb and FG medullary injections were focussed on the LRt but a penumbra of diffuse staining which encroached upon the intermediate and parvicellular reticular nuclei was present in all three animals (**Figures 1 and 2**). Injections of CTb and FG labeled cells on both sides of the gray matter in all three spinal segments were analyzed (**Table 1**, Experiment 1). The total numbers of SRT cells counted for each animal ranged from 656 to 796 but FG injections produced greater numbers of cells (63% of all cells labeled). The largest numbers of cells were found contralateral to their respective injection sites: 56% (±6.38 SD) of CTb-labeled cells and 61% (±9.9 SD) of FG-labeled cells. However, small numbers of cells on both sides of the gray matter were double-labeled for FG and CTb: 10% (±4.7 SD) and 7% (±2.87 SD) on the right and left sides of sections, respectively (**Figure 3**). The majority of cells, both contralateral and ipsilateral to injection sites were located within medial areas of lamina VI and VII and within lamina X. Additional populations were found within the reticulated region of lamina V and small numbers were present in lamina I. In the L5 segment, cells were also present in Lamina VIII (see **Figure 2**).

### SPINAL INJECTION SITES AND DISTRIBUTION OF TERMINALS IN THE LRt

**FIGURE 4:**
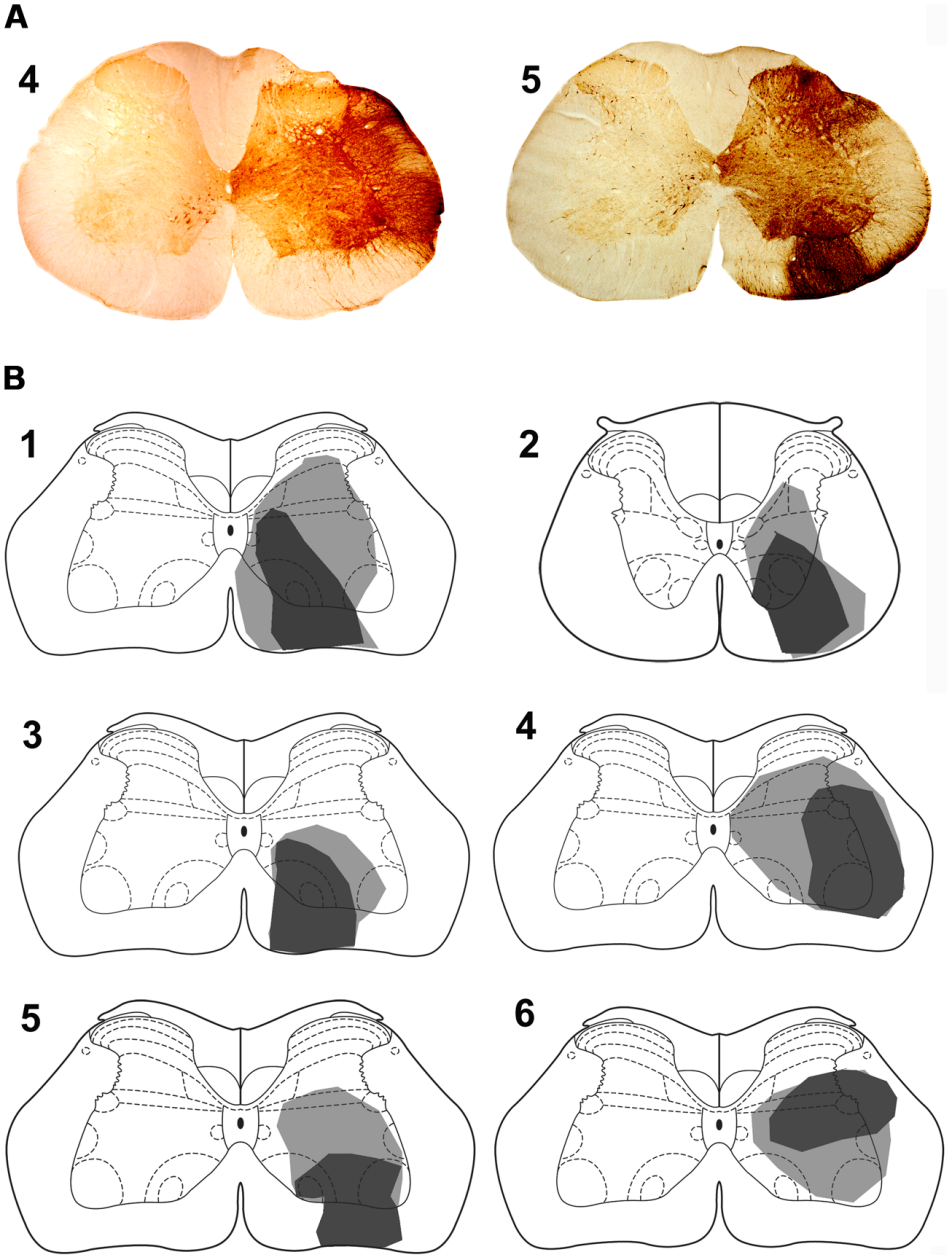
**Spinal injection sites.** **(A)** Photomicrograps of the L4 spinal segment showing CTb injection site for animals 4 and 5. The drawings **(B)** show the spread of CTb within the gray and white matter. The dark region represents the core of the injection and the gray region the spread of CTb. Spinal injections are in L4 with the exception of animal 2 which was in T11. Note that the core of most injections is within the ventral quadrant of the cord except for animal 6 where it was in the intermediate gray matter. Animals 1–3 were also used in experiments to label precerebellar cells.

**FIGURE 5:**
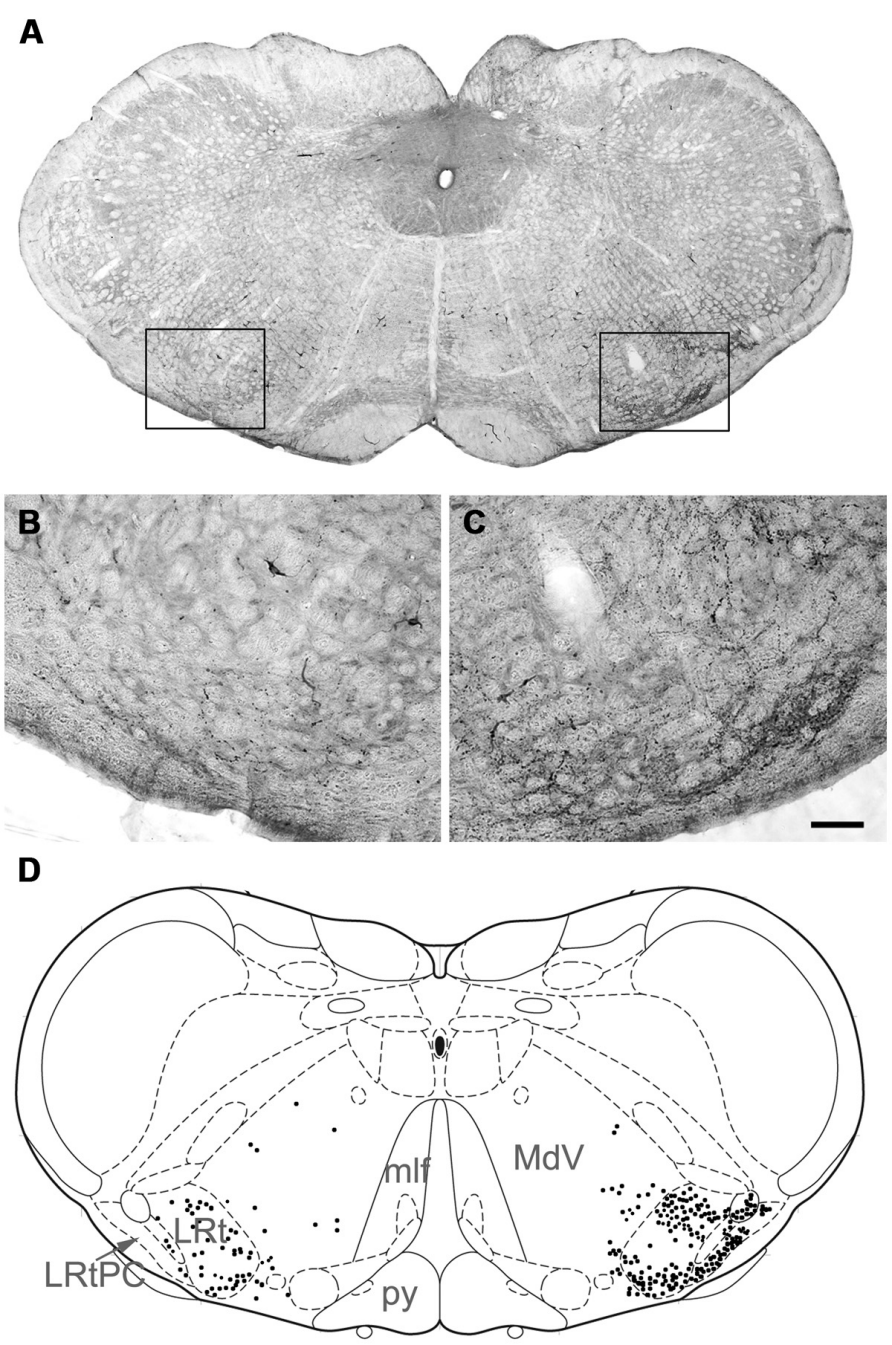
**(A)** A photomicrograph showing a coronal section through the medulla the areas demarcated by boxes on the left and the right are shown at high magnification in **(B)** and **(C),** respectively, which show terminal labeling within the LRt contralateral and ipsilateral to the spinal injection site. Note the particularly large numbers of terminals in the ipsilateral LRt. **(D)** A representation of **A** (based on Paxinos and Watson, 2005) with terminal locations plotted to show their relationships with medullary structures (right = contralateral to spinal injection; -14.4 relative to Bregma). LRt, lateral reticular nucleus; LRtPC lateral reticular nucleus, parvicellular part; MdV, medullary reticular nucleus; mlf, medial longitudinal fasciculus; py, pyramid. Scale bar for **(B,C)** = 100 μm.

For five animals (animals 1 and 3–6), injections of CTb were within the L4 segment but for one animal the injection was located in T11 (animal 2). For animals 1–5, CTb labeling was most intense within the right ventrolateral to ventromedial regions of segments L1–4 but CTb spread throughout the ventral horn and extended into the intermediate gray matter but there was no spread to the left side of the cord (**Figure 4**). In one animal (animal 6) the injection was concentrated within the intermediate gray matter (**Figure 4**) but there was spread of CTb into the ventral horn and ventral white matter. In all animals, large numbers of anterogradely labeled axons were concentrated in the LRt ipsilateral to the spinal injection and considerably fewer terminals were present in the contralateral side (**Figure 5**). The ratio of ipsilateral versus contralateral terminals was 2.6 to 1. In the rat, the LRt extends from interaural co-ordinate -4.32 to -6.00 mm (Paxinos and Watson, 2005) but the largest regions are found between -4.68 to -5.16 mm and it is in this region that the greatest concentration of labeled terminals was observed. The concentration of terminals progressively declines more rostrally especially in the magnocellular areas. Mediolaterally the distribution follows the various shapes of the LRt but the greatest terminal density is found in the intermedio-medial area. Caudally, terminals were most concentrated in ventral regions of the LRt especially within the parvicellular part of the nucleus.

### IMMUNOCHEMICAL PROPERTIES OF SRT AXON TERMINALS IN THE LRt

**FIGURE 6:**
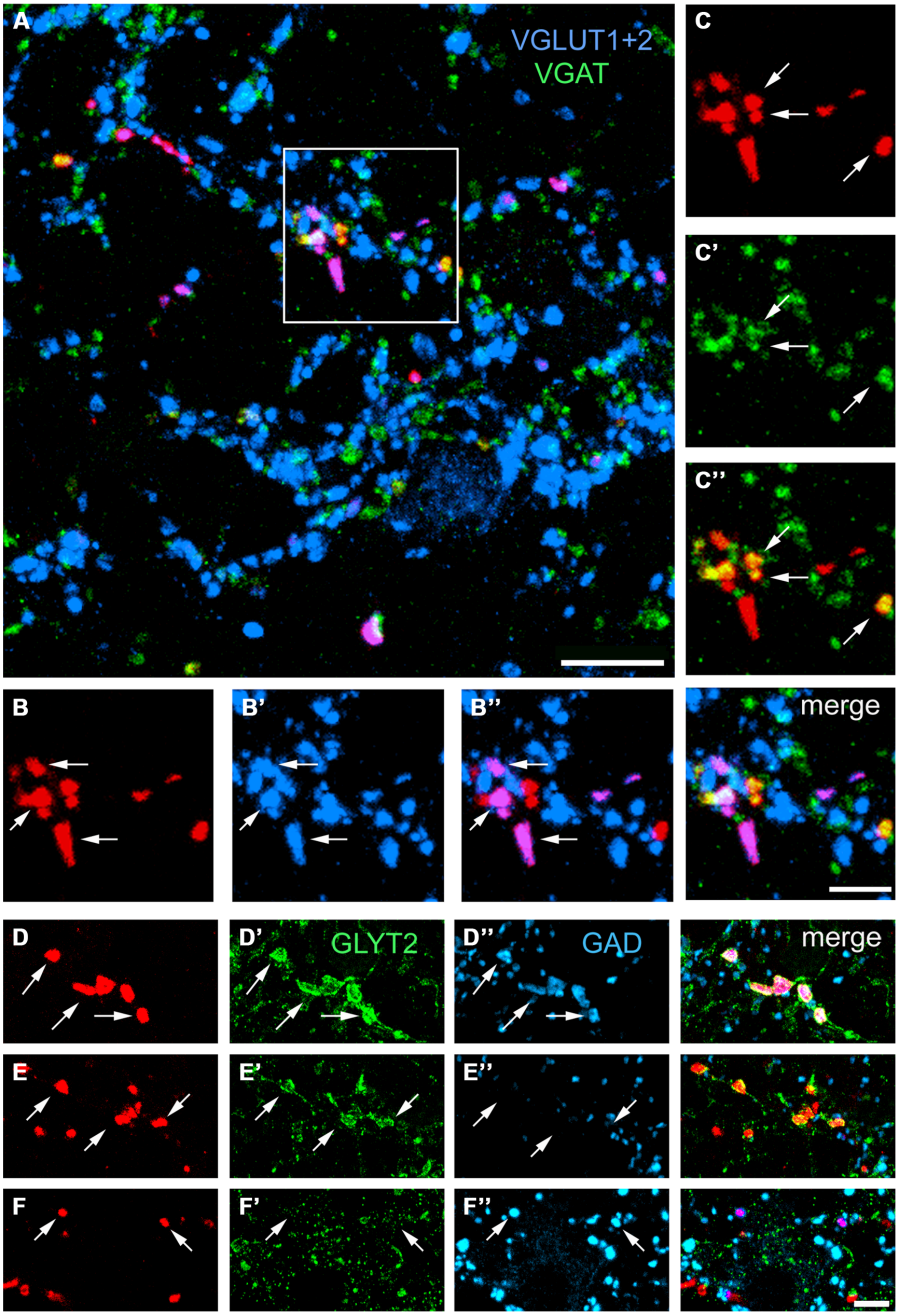
**Single optical sections showing immunochemical properties of axon terminals projecting from the lumbar spinal cord in the lateral reticular nucleus ipsilateral to the spinal injection.** **(A)** An overview of terminals within the LRt labeled for CTb, VGLUT1+2, and VGAT. The area demarcated by the box is shown in series **(B,C)**. **(B)** CTb terminals (red) that are labeled for VGLUT1+2 **(B’,B”)**. **(C)** CTb terminals that are labeled for VGAT **(C’,C”)**. A merged image from both series is shown on the right adjacent to **(B”)**. **(D)** CTb terminals that are labeled for GlyT2 **(D’)** and GAD **(D”)**. **(E)** Terminals that are labeled for GlyT2 **(E’)** but not GAD **(E”)**. **(F)** Terminals that are not labeled for GlyT2 **(F’)** but are labeled for GAD **(F”)**. Arrows indicate double-labeled structures in each series. VGLUT1+2, vesicular glutamate transporter 1 and vesicular glutamate transporter 2; VGAT, vesicular GABA transporter; GLYT2, glycine transporter 2; GAD glutamate decarboxylase. Scale bars **(A)** = 10 μm, **(B,C)** = 5 μm, **(D–F)** = 5 μm.

Confocal microscope images of SRT axon terminals in the ipsilateral and contralateral LRt labeled according to the procedures in **Table 12A–C** are shown in **Figure 6**. Data concerning these experiments are provided in **Table 2**. To determine the percentages of excitatory and inhibitory SRT terminals in the LRt, sections were reacted to reveal CTb, VGAT and a combination of VGLUT1 and 2 (Experiment 2A, **Table 1**). In total 4401 CTb terminals were counted in the side ipsilateral to the injection site and although most of the terminals were immunoreactive for VGLUTs (76.9 ± 3.0%: mean ± SD%) a substantial number was reactive for VGAT (20.1 ± 3.1%). In order to clarify what types of VGLUTs are present in CTb-labeled terminals in the LRt, sections were reacted to reveal CTb, VGLUT1 and VGLUT2 (Experiment 2B, **Table 1**). A total of 2410 terminals was examined through stacks of tile scans of the LRt. Analysis of these scans revealed that 74.9 (±2.8) % of the terminals was immunopositive for VGLUT2 but only a very small percentage (1.8 ± 0.6%) of terminals was immunoreactive for VGLUT1 (**Table 2**). A total of 2732 CTb-labeled terminals was examined to investigate further the nature of the inhibitory subpopulation (Experiment 2C, **Table 1**). Analysis of confocal microscope images (**Figures 6D–F**) revealed that the percentage of inhibitory terminals labeled by GAD, GLYT2 or with both GAD and GLYT2 was 4.9 ± 0.5, 12.7 ± 2.0% and 4.9 ± 1.0%, respectively, of the total numbers of CTb-labeled terminals). There was no obvious segregation of CTb terminals labeled with VGLUT and VGAT as both types of terminal were present in overlapping areas within the LRt.

**Table 2:** Percentages of immunoreactive terminals in the ipsi- and contralateral lateral reticular nuclei anterogradely labeled from spinal injections of the b subunit of CTb into L3 and 4 segments.

Ipsilateral						Contralateral					
Animal	Terminals	VGLUT1&2	VGAT	VGLUT1/2 &VGAT	CTb	Animal	Terminals	VGLUT1&2	VGAT	VGLUT1/2 & VGAT	CTb
1	768	77.5	18.4	1.7	2.5		1	189	73.5	22.8	1.1	2.6
2	801	73.8	23.3	1.1	1.7		2	439	74.5	22.8	0.9	1.8
3	853	76.1	21.6	0.8	1.5		3	238	76.5	20.2	1.3	2.1
4	498	79.5	17.9	0.2	2.4		4	147	73.6	21.7	0.8	3.9
5	755	73.6	23.6	0.7	2.1		5	363	73.2	23.4	0.3	3.1
6	726	80.9	16.1	1.4	1.7		6	315	81.6	15.9	1.6	1.0
	**Mean%**	**76.9**	**20.1**	**1.0**	**2.0**			**Mean%**	**75.5**	**21.1**	**1.0**	**2.4**
	±SD	3.0	3.1	0.5	0.4			±SD	3.2	2.8	0.4	1.0
												
		**VGLUT1**	**VGLUT2**	**VGLUT1&2**	**CTb**				**VGLUT1**	**VGLUT2**	**VGLUT1&2**	**CTb**
1	391	2.0	77.5	0.5	19.9		1	84	1.2	78.6	1.2	19.0
2	396	1.3	71.5	1.3	26.0		2	215	0.9	77.7	0.9	20.5
3	389	1.8	73.5	0.3	24.4		3	123	0.8	82.1	0.0	17.1
4	174	2.9	75.9	2.9	18.4		4	167	1.2	72.5	0.6	25.7
5	359	1.1	72.7	0.8	25.3		5	194	2.1	74.7	2.1	21.1
6	701	2.0	78.5	0.6	19.0		6	230	2.6	74.3	3.0	20.0
	**Mean%**	**1.8**	**74.9**	**1.1**	**22.2**			**Mean%**	**1.5**	**76.7**	**1.3**	**20.6**
	±SD	0.6	2.8	1.0	3.4			±SD	0.7	3.5	1.1	2.9
												
		**GAD**	**GLYT2**	**GAD&GLYT2**	**CTb**				**GAD**	**GLYT2**	**GAD&GLYT2**	**CTb**
1	490	4.9	14.5	5.9	74.7		1	193	7.3	10.4	6.2	76.2
2	408	4.7	15.0	4.9	75.5		2	258	6.2	10.9	5.0	77.9
3	363	4.7	12.5	5.0	77.7		3	89	4.5	13.5	5.6	76.4
4	403	4.5	12.7	3.0	79.9		4	257	10.1	10.1	2.7	77.0
5	541	5.9	12.4	5.0	76.7		5	318	7.2	12.9	4.1	75.8
6	527	4.7	9.3	5.5	80.5		6	309	7.4	10.0	4.9	77.7
	**Mean%**	**4.9**	**12.7**	**4.9**	**77.5**			**Mean%**	**7.1**	**11.3**	**4.8**	**76.8**
	±SD	0.5	2.0	1.0	2.3			±SD	1.8	1.5	1.2	0.9

*VGLUT1, vesicular glutamate transporter 1; VGLUT2, vesicular glutamate transporter 2; VGAT, vesicular GABA transporter; GLYT2, glycine transporter 2; GAD glutamate decarboxylase. VGLUT1+2, tissue incubated in a mixture of both antibodies, CTb, terminals only immunoreactive for the b subunit of cholera toxin.*

Although fewer terminals were present within the LRt contralateral to the injection site, the pattern of immunoreactivity was similar to the ipsilateral LRt (**Table 2**). For combined VGLUT1 and 2 antibodies, 75.5 ± 3.2% of terminals was positively labeled whereas 21.1 ± 2.8% was labeled for VGAT. Similar numbers of contralateral terminals were labeled for VGLUT 2 in Experiment 2A (74.9 versus 76.7%) and for Experiment 2C terminals labeled for GAD, GLYT2, and both markers were present in similar proportions to the ipsilateral side. None of these values were significantly different from the ipsilateral side (ANOVA).

### ANALYSIS OF CONTACTS MADE BY SRT AXON TERMINALS AND PRE-CEREBELLAR NEURONS IN THE LRt

**FIGURE 7:**
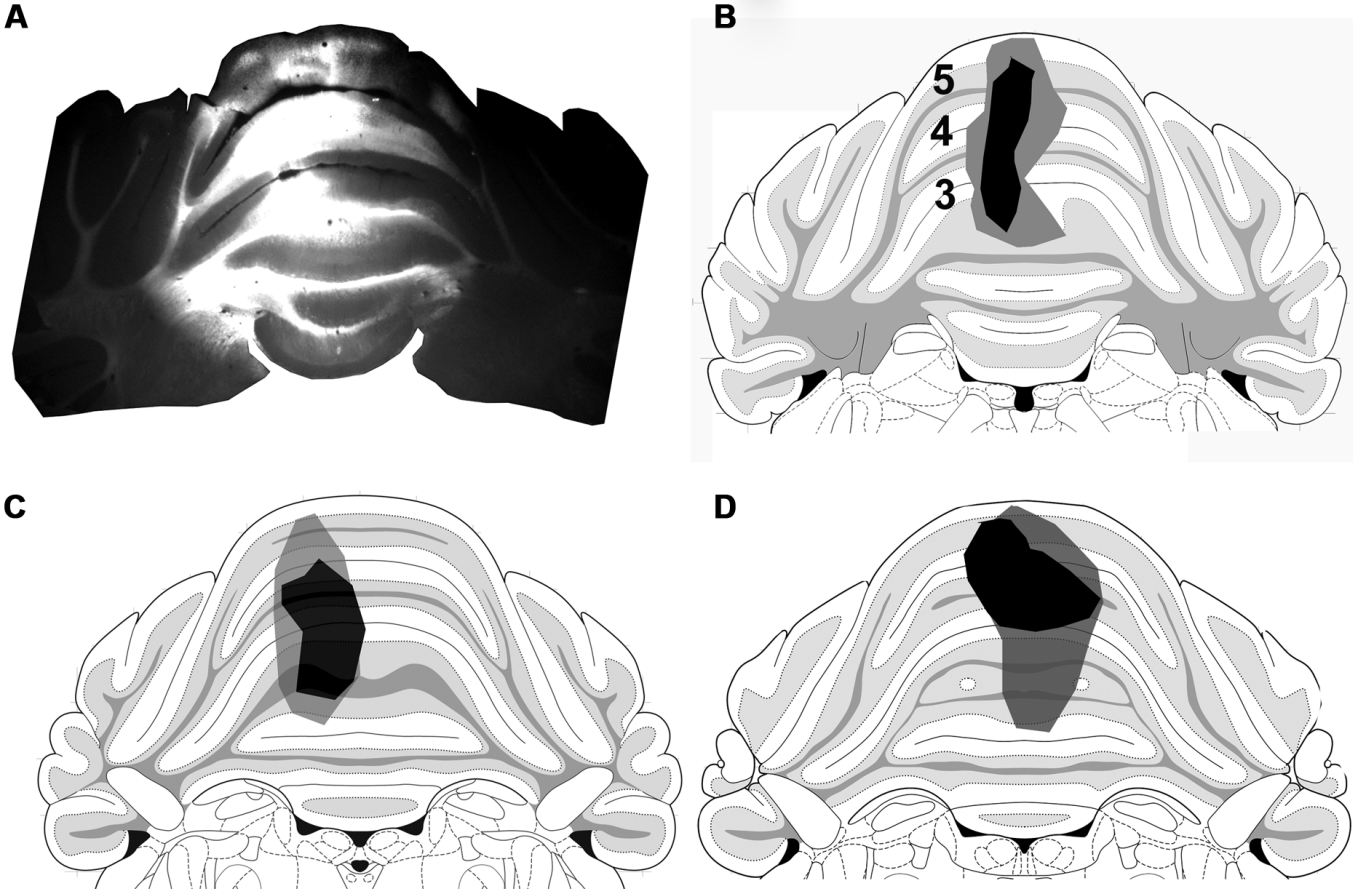
**Cerebellum injection sites.** **(A)** A fluorescence micrograph showing a FG injection site within a coronal section of the anterior cerebellum. **(B–D)** Drawings of the core (black) and spread (gray) of FG within lobules 3, 4, and 5 of animals 1–3, respectively, (based on Paxinos and Watson, 2005).

**FIGURE 8:**
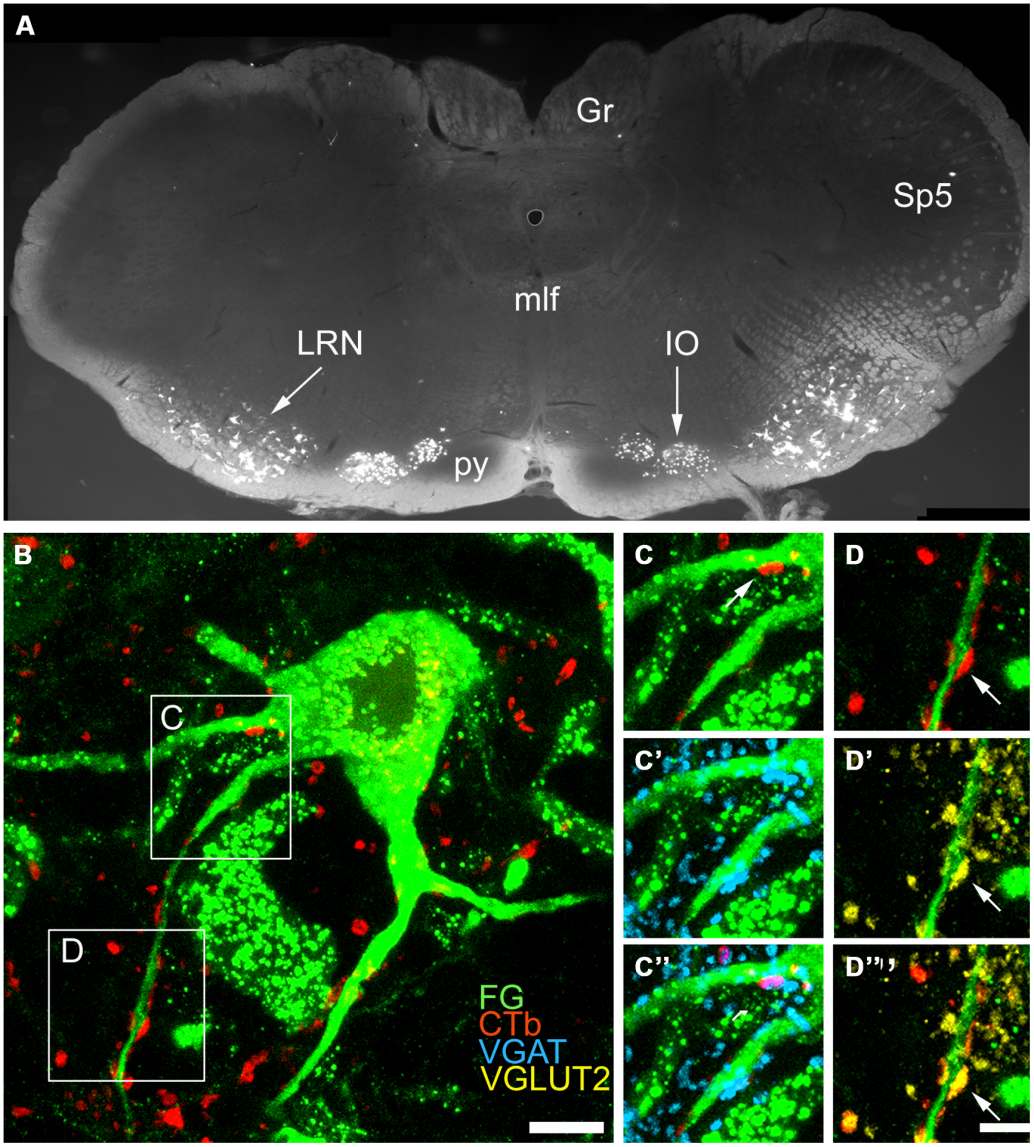
**Immunochemical properties of contacts on pre-cerebellar neurons in the LRt.** **(A)** A fluorescent micrograph showing a coronal section through the medulla containing pre-cerebellar neurons labeled with FG from the anterior cerebellum. Note the cells within the LRt and the inferior olivary complex (IO). (Gr, Gracile nucleus; mlf, medial longitudinal fasciculus; py, pyramid; Sp5, spinal trigeminal nucleus). **(B)** A single optical section showing a precerebellar neuron (green) with several CTb (red) contacts on it. The contact shown in the box **(C)** is immunoreactive for VGAT **(C–C”)** and the contact shown in box **(D)** is immunoreactive for VGLUT2 **(D–D”)**. CTb, b subunit of cholera toxin; FG, Fluorogold; VGLUT2, vesicular glutamate transporter 2; VGAT, vesicular GABA transporter. Scale Bars **(B)** = 10 μm; **(C**,**D)** = 5 μm.

In these experiments we examined contacts from the ipsilateral SRT only. FG injections were made into the anterior lobe of the cerebellum (Focused on lobules III, IV, V) and injections of CTb were made into the right side of lumbar spinal segments (**Figure 7**). Injections of FG in the cerebellum resulted in extensive labeling of pre-cerebellar neurons in the medulla which were mainly confined to the LRt and inferior olivary complex (**Figure 8A**). Diameters of neurons labeled within the LRt ranged from 18 to 33 μm. Relationships between ipsilateral SRT terminals and pre-cerebellar neurons in the LRt were assessed by using four channel confocal imaging. Sections were processed to reveal CTb, FG, VGLUT2, and VGAT (Experiment 3, **Table 1**; see **Figures 8B–D**). Of all immunoreactive terminals contacting pre-cerebellar neurons, 54% were VGAT immunoreactive whereas 46% contained VGLUT2; however, 29% of all excitatory contacts on LRt cells originate from the lumbar spinal cord but less than 6% of inhibitory terminals are of spinal origin. Seventeen percent of all contacts on LRt cells were made by spinoreticular terminals but most of these CTb-labeled contacts were VGLUT2 positive (80%) whereas a minority were VGAT-immunoreactive (19%). The average number of CTb-VGLUT2 terminals contacting LRt cells was 21.6 (±10.6 SD) and the average number of CTb–VGAT terminals was 5.1 (±1.6 SD). The average contact density for excitatory SRT terminals cells was found to be 0.14 (±0.10 SD) and 0.59 (±0.38 SD) per 100 μm^2^ for somata and dendrites, respectively, whereas the average density for inhibitory terminals was only 0.08 (±0.05) for somata and 0.12 (±0.02 SD) per100 μm^2^ for dendrites. Full details of contact densities on cell bodies and dendrites are given in **Table 3**. All cells analyzed received contacts from VGLUT2 containing SRT terminals and 87% of them received convergent inputs from both inhibitory and excitatory spinal axons.

**Table 3:** Immunochemical properties of contacts on pre-cerebellar cells within the lateral reticular nucleus.

Animal	VGLUT2	VGAT	VGLUT2/CTb	VGAT/CTb	CTb
**SOMA**
1	0.40	0.99	0.09	0.08	0.02
2	0.59	1.01	0.09	0.03	0.00
3	0.84	1.04	0.26	0.13	0.01
**Mean**	**0.61**	**1.01**	**0.14**	**0.08**	**0.01**
SD	0.22	0.03	0.10	0.05	0.01
**DENDRITE**
1	1.02	1.40	0.54	0.13	0.05
2	1.83	3.71	0.99	0.13	0.04
3	1.39	1.38	0.23	0.10	0.00
**Mean**	**1.35**	**2.05**	**0.59**	**0.12**	**0.04**
SD	0.41	1.34	0.38	0.02	0.03

*The table shows average contact packing densities per 100 μm^*2*^ for contacts on somata and dendrites for three animals. Eleven cells were examined from each animal. VGLUT2, terminals immunoreactive vesicular glutamate transporter 2 alone; VGAT, terminals immunoreactive vesicular GABA transporter alone; VGLUT2/CTb, VGLUT2 terminals immunoreactive for both CTb and VGLUT 2; VGAT/CTb, terminals immunoreactive for both CTb and VGAT; CTb, terminals only immunoreactive for the b subunit of cholera toxin.*

## DISCUSSION

In the present study it was found that projections from the lumbar spinal cord terminate mostly within the ipsilateral LRt with fewer projections terminating within the contralateral LRt. This observation seems paradoxical as findings from the present and previous studies indicate that the majorly of SRT cells in the lumbar cord project to the contralateral LRt. A probable explanation for this finding is discussed below. In addition, bilaterally projecting SRT cells were also observed which were present principally within the intermediate gray matter. Our findings were similar to those of Garifoli et al. (2006) who reported that roughly 7% of SRT cells with bilateral projections were located mainly in laminae III, IV, VII, and VIII. This is in contrast to the findings of Koekkoek and Ruigrok (1995) who reported that very small numbers (2%) of cells with bilateral projections were present only within the dorsal horn. Lumbar spinal projections to the LRt are predominantly excitatory but there is a significant inhibitory component that consists of three subtypes of axon containing GABA, glycine, or a mixture of GABA and glycine. Similar proportions of excitatory and inhibitory terminals were found in ipsilateral and contralateral projections. The overwhelming majority of ipsilateral SRT terminals (80%) form excitatory contacts with LRt pre-cerebellar neurons and smaller numbers of inhibitory contacts were also formed (∼19%). Many cells received convergent connections from excitatory and inhibitory axons.

### TECHNICAL CONSIDERATIONS

In this study CTb was used as an anterograde tracer (see Ericson and Blomqvist, 1988) to label spinal axons that project to the LRt. CTb is not only taken up by neuronal cell bodies but it is also taken up by axons of passage (Chen and Aston-Jones, 1995). Most of our spinal injection sites were focussed upon the ventrolateral/ventromedial region of the cord but there was spread also into the intermediate gray matter. The present and previous studies of retrogradely labeled spinal cells following injections of tracer substances into the LRt (Corvaja et al., 1977a; Hrycyshyn and Flumerfelt, 1981; Menétrey et al., 1983; Shokunbi et al., 1985; Koekkoek and Ruigrok, 1995; Lee and Mihailoff, 1999) reveal that neurons in laminae VII, VIII, and X project predominantly to the contralateral LRt (Menétrey et al., 1983; Shokunbi et al., 1985). The axons of bVFRT neurons ascend in the ventral part of the lateral funiculus (Ekerot, 1990b) and project contralaterally at the segmental level (Holmqvist et al., 1960; Lundberg and Oscarsson, 1962; Rosen and Scheid, 1973a; Ekerot, 1990b). Thus a possible explanation of why spinal-LRt projections were found to be mainly to the ipsilateral LRt in our study is that CTb was principally taken up and transported by axons of passage within the gray and white matter. This conclusion is also supported by the classical observations of Brodal (1949) in the cat who used the Marchi method in combination with spinal lesions and more recent observations of Rajakumar et al. (1992) who found predominant ipsilateral projections to the LRt following unilateral injections of HRP-wheatgerm agglutinin within rat cervical segments. Although many of the labeled axon terminals could belong to the bVFRT, it is not possible to conclude that they originate exclusively from this tract as some injections encompassed more medial regions of the ventral gray and white matter and yet significant projections to the LRt with similar neurochemical profiles were observed.

An additional limitation of the study is that the FG tracing method used to label pre-cerebellar LRt cells does not label entire dendritic trees of neurons and therefore the numbers of excitatory and inhibitory contacts on these cells is likely to have been underestimated as we were only able to produce data regarding contact packing densities associated with somata and first and second order dendrites. Although we cannot be certain that SRT terminals contacting LRt cells form synapses, the presence of vesicular transporters within them confirms that they contain synaptic vesicles; therefore it is likely that many of the contacts observed do represent synaptic associations. Furthermore, studies using combined confocal and electron microscopy (e.g., Todd, 1997; Olave and Maxwell, 2003) along with confocal microscopic studies using markers to label postsynaptic densities (e.g., Shakya Shrestha et al., 2012) confirm that the vast majority of contacts observed with confocal microscopy between axonal swellings and cells are indeed synaptic. Finally even though our sample of cells was small (33 in total) we were able to detect consistent contact patterns in cells from all three animals; for example almost all cells in the sample had convergent contacts from excitatory and inhibitory SRT axons but in most cases excitatory contacts greatly outnumbered inhibitory ones.

### SRT PROJECTIONS TO THE LRt

Previous observations on the rat and cat fit well with our findings showing that lumbar projections were located in ventral and lateral regions of the nucleus (Brodal, 1949; Rajakumar et al., 1992). The first studies of spinal projections to the LRt were made by Brodal (1949) using Glees’ silver method in adult cats that had received lesions of the cervical and lumbar white matter. This study showed that the principal spinal projections to the LRt travel in the lateral funiculus and revealed that terminations were organized somatotopically within the nucleus. Many spinal projections to the LRt terminate in the parvicellular region and projections from lumbar regions were found predominantly within ventrolateral regions whereas cervical projections were most abundant within dorsomedial regions. Rajakumar et al. (1992) used wheatgerm agglutinin conjugated to HRP in a study of afferent projections to the LRt in rats. Lumbar injections resulted in terminal labeling in medial and ventrolateral regions of the LRt and encompassed both magnocellular and parvicellular components of the nucleus. A somatotopic arrangement of afferent input to the LRt is consistent with the electrophysiological observations of Ekerot (1990b) who noted that LRt cells activated by the bVFRT were located in ventrolateral regions of the nucleus. Further evidence for a somatotopic arrangement within the nucleus in the mouse was provided in a recent report using viral tracing methods (Pivetta et al., 2014).

### INHIBITORY AND EXCITATORY TERMINALS IN THE LRt

Ekerot (1990b) provided evidence that electrical stimulation of the ventrolateral quadrant could evoke monosynaptic EPSPs and IPSPs in LRt cells and responses indicated that excitatory and inhibitory SRT neurones terminated in overlapping areas. In this study we have shown that spinal axons within the LRt contain markers for inhibitory and excitatory neurotransmitters and that there is no obvious segregation of excitatory and inhibitory termination sites of axons originating from the lumbar cord (cf. Pivetta et al., 2014). Also inhibitory and excitatory SRT axons form contacts with pre-cerebellar LRt neurons projecting to the anterior lobe of the cerebellum and some cells had convergent contacts form both types of axon. However, Ekerot (1990b) on the basis of electrophysiological evidence concluded that the bVFRT consisted of roughly equal groups of excitatory and inhibitory neurons whereas we found that excitatory contacts form a significant proportion (29%) of excitatory inputs to these cells whereas inhibitory SRT axons constitute only 6% of inhibitory contacts. The reasons for this apparent discrepancy are unclear, they could be a consequence of the different methodological approaches used (see technical issues above) or may represent a species difference between cats and rats.

Our study indicates that there may be three distinct groups of inhibitory systems projecting to the LRt from the spinal cord; one that is exclusively glycinergic, one that is exclusively GABAergic and one that contains a mixture of both transmitters. Pharmacological information on the LRt is apparently very limited but ligand binding, *in situ* hybridization and immunocytochemical studies show that cells of the LRt express GABA and glycine receptors (Gale et al., 1980; Araki et al., 1988; Sato et al., 1991). However, it is not known how these receptors are organized and whether they are expressed by all LRt neurons or only a subpopulation of cells. Nevertheless it is probable that these subgroups of inhibitory cells exert subtly different effects on their LRt target neurons. In addition to terminals originating from the lumbar spinal cord, pre-cerebellar neurons in the ventral and lateral regions of the LRt have an abundance of excitatory and inhibitory contacts on them. All of these terminals originate from sources within the spinal cord and brain as the LRt contains no interneurons (Kapogianis et al., 1982a,b).

### FUNCTIONAL IMPLICATIONS

The LRt is a complex structure which receives multiple inputs from different lumbar and cervical spinal systems and various brain structures such as the primary motor cortex, red nucleus, and superior colliculus. Cells of the LRt project widely to different regions of the cerebellum and deep cerebellar nuclei. Some of these input/output systems appear to be segregated. As discussed above, spinal terminations within the LRt are arranged somatotopically and this somatotopic organization is conserved by LRt projections to the cerebellum (e.g., see Alstermark and Ekerot, 2013). Most LRt cells receive convergent inputs from different spinal and brain systems (e.g., see Kitai et al., 1974) and therefore the LRt has an integrative function. The cells forming SRT projections to the LRt are located principally in laminae VI, VII, and X where many last order premotor interneurons are located (Tripodi et al., 2011) and therefore one of the functions of this pathway may be to monitor activity of such interneurons. In addition, some individual SRT cells have the capacity to directly influence activity of cells within both the left and right LRt. Thus the spino-bulbar-reticular pathway appears to provide the cerebellum with information that is different and distinct from classical spinocerebellar pathways. The cells of origin of the pathway are located more ventrally to those of the dorsal spinocerebellar pathway and more medially to the cells of the ventral spinocerebellar pathway. Information from the lumbar spinal cord is integrated with vestibular input at the spinal level and with input from a variety of supraspinal sources within the LRt itself. Thus the cerebellum appears to use information provided by the classical ‘pure lines’ from the spinal cord and compare it with information provided via the indirect pathway in order to make motor adjustments to maintain normal posture during locomotor and other motor behaviors (e.g., see Alstermark and Ekerot, 2013).

## Conflict of Interest Statement

The authors declare that the research was conducted in the absence of any commercial or financial relationships that could be construed as a potential conflict of interest.

## ABBREVIATIONS

bVFRT: bilateral ventral flexor reflex tract
CTb: cholera toxin B subunit
DAB: 3,3′-diaminobenzidine
GAD: glutamic acid decarboxylase
GLY T2: glycine transporter 2
HRP: horseradish peroxidise
iFT: ipsilateral forelimb tract
IgG: immunoglobulin gamma
LRt: Lateral reticular nucleus
PB: Phosphate buffer
PBS: Phosphate buffer saline
PBST: phosphate buffer saline containing 0.3% Triton X-100
SD: standard deviation
SRT: Spinoreticular tract
VGAT: vesicular GABA transporter
VGLUT: vesicular glutamate transporter
